# G Protein-Coupled Receptor 87 (GPR87) Promotes the Growth and Metastasis of CD133^+^ Cancer Stem-Like Cells in Hepatocellular Carcinoma

**DOI:** 10.1371/journal.pone.0061056

**Published:** 2013-04-10

**Authors:** Mingxia Yan, Hong Li, Miaoxin Zhu, Fangyu Zhao, Lixing Zhang, Taoyang Chen, Guoping Jiang, Haiyang Xie, Ying Cui, Ming Yao, Jinjun Li

**Affiliations:** 1 State Key Laboratory of Oncogenes and Related Genes, Shanghai Cancer Institute, Renji Hospital, Shanghai Jiaotong University School of Medicine, Shanghai, China; 2 Qi Dong Liver Cancer Institute, Qi Dong, Jiangsu Province, China; 3 Department of General Surgery, The First Affiliated Hospital, School of Medicine, Zhejiang University, Hangzhou, China; 4 Cancer Institute of Guangxi, Nanning, China; The University of Hong Kong, China

## Abstract

Hepatocellular carcinoma (HCC) is a prevalent disease worldwide, and the majority of HCC-related deaths occur due to local invasion and distant metastasis. Cancer stem cells (CSCs) are a small subpopulation of cancer cells that have been hypothesized to be responsible for metastatic disease. Recently, we and others have identified a CSC population from human HCC cell lines and xenograft tumors characterized by their expression of CD133. However, the precise molecular mechanisms by which CD133^+^ cancer stem-like cells mediate HCC metastasis have not been sufficiently analyzed. Here, we have sorted HCC cells using CD133 as a cancer stem cell (CSC) marker by magnetic-activated cell sorting (MACS) and demonstrated that the CD133^+^ HCC cells not only possess greater migratory and invasive capacity *in vitro* but are also endowed with enhanced metastatic capacity *in vivo* and in human HCC specimens when compared to CD133^−^ HCC cells. Gene expression analysis of the CD133^+^ and CD133^−^ cells of the HCC line SMMC-7721 revealed that G protein-coupled receptor 87 (GPR87) is highly expressed in CD133^+^ HCC cells. In this study, we explored the role of GPR87 in the regulation of CD133 expression. We demonstrated that the overexpression of GPR87 up-regulated CD133 expression, promoted CSC-associated migratory and invasive properties *in vitro*, and increased tumor initiation *in vivo*. Conversely, silencing of GPR87 expression reduced the levels of CD133 expression. Conclusion: GPR87 promotes the growth and metastasis of CD133^+^ cancer stem-like cells, and our findings may reveal new targets for HCC prevention or therapy.

## Introduction

Hepatocellular carcinoma (HCC) is one of the most common human cancers, and the majority of HCC-related deaths occur due to local invasion and distant metastasis [1]. Despite significant progress, most therapeutic approaches fail to eliminate all tumor cells, and the residual cancer cells often result in tumor recurrence and metastasis. Increasing evidence has shown that cancer stem cells (CSCs) may be responsible for resistance to conventional therapy and metastatic disease [2], [3], [4], [5]. However, the molecular mechanisms of metastasis are not sufficiently understood.

CD133, a 5-transmembrane glycoprotein, is an important cell surface protein that has been identified as a cancer stem cell marker in various solid tumors [6], [7], [8], including liver cancer [5], [9], [10]. Our previous study first identified and confirmed the existence of a small subpopulation of CD133^+^ HCC cells within HCC cell lines that exhibited increased clonogenicity *in vitro* and potent tumorigenicity *in vivo* [11]. Other groups have also demonstrated that CD133^+^ HCC cells possess cancer stem cell-like properties, including self-renewal, differentiation, *in vivo* tumor initiation and chemotherapy resistance [12], [13], [14], [15]. However, little is known about the role of CD133^+^ HCC cells in tumor metastasis.

G protein-coupled receptor 87 (GPR87), also known as GPR95, is a cell surface GPR that is overexpressed in diverse cancers and plays an essential role in tumor cell survival [16], [17]. Although much evidence suggests that GPRs play important roles in the regulation of cell morphology, polarity and migration [18], [19], [20], there are few reports about the function of GPR87. Only two reports have shown that GPR87 knockdown sensitized cancer cells to DNA damage–induced growth suppression via enhanced p53 stabilization and activation [16], [21].

In the present study, we isolated a CD133^+^ CSC-like subpopulation from human HCC cell lines and demonstrated that the CD133^+^ HCC cells displayed migratory and invasive properties *in vitro* and possessed metastatic potential *in vivo*. Moreover, we explored the role of GPR87 in regulating the expression of CD133, which in turn promoted the growth and metastasis of cancer stem-like cells in HCC.

## Materials and Methods

### Cell Culture

The Hep3B, SNU475 and PLC/PRF/5 HCC cell lines were purchased from the American Type Culture Collection (ATCC) (Manassas, VA, USA). The SMMC-7721 cell line was obtained from the Cell Bank of the Institute of Biochemistry and Cell Biology, China Academy of Sciences (Shanghai, China). The MHCC-97L line was provided by the Liver Cancer Institute of Zhongshan Hospital, Fudan University (Shanghai, China). The HCC-LY5 cell line was established in our laboratory by isolation from a HCC sample from a patient who had undergone liver cancer resection in the Liver Cancer Institute of Zhongshan Hospital, Fudan University (Shanghai, China). All cell lines were cultured in Dulbecco's Modified Eagle's Medium (DMEM; Sigma-Aldrich, USA) containing 10% heat-inactivated FBS (Biowest, France) supplemented with 100 I.U./ml penicillin G and 100 µg/ml streptomycin (Sigma-Aldrich, USA), and the cells were incubated at 37°C in a humidified atmosphere with 5% CO_2_.

### Cell Isolation by Magnetic-Activated Cell Sorting (MACS)

The cells were labeled with primary CD133/1 antibody (mouse IgG1, Miltenyi Biotec, Germany), and the CD133^+^ and CD133^−^ cells were subsequently magnetically isolated using the EasySep PE Selection Kit (StemCell Technologies, Canada) according to the manufacturer's instructions. The purity of the sorted cells was evaluated by Western blot. Trypan blue staining was used to assess the sorted cell viability, and greater than 90% viability was considered acceptable for downstream experiments.

### Lentivirus Production and Cell Transduction

The GPR87 ORF sequence was PCR-amplified using specific primers (forward: 5′- TCGACGCGTATGGGGTTCAACTTGACGCTTG-3′, reverse: 5′- TTCCATATGGGCAAACATTACACGCAGACAA-3′) and cloned into the pWPXL lentiviral expression vector (Addgene, MA) by replacing the GFP fragment. The CD133 cDNA clone (Myc-DDK-tagged ORF clone of Homo sapiens prominin 1 (PROM1), transcript variant 1 as transfection-ready DNA NM_006017.1) with full length ORF sequence was purchased from Origene (OriGene Technologies, Inc. Rockville), which was cloned into the pWPXL lentiviral expression vector (Addgene, MA) by replacing the GFP fragment. The pLVTHM-shGPR87 and pLVTHM-shNC vectors were constructed by inserting annealed oligos (GPR87: 5′- CCGGUGCUUUAUCUCAUUATT-3′ or 5′- GGACCUUGGUACUUCAAGUTT-3′, NC: 5′- TTCTCCGAACGTGTCACGT-3′) into the lentiviral pLVTHM vector as described on the Addgene website.

The viral packaging was performed in HEK 293T cells after co-transfection of the pWPXL-GPR87 or pLVTHM-shGPR87 vector with the psPAX2 packaging plasmid and the pMD2.G envelope plasmid (Addgene, MA) using Lipofectamine 2000 (Invitrogen, Canada). The viruses were harvested 72 h after transfection, and the viral titers were determined. Target cells (1×10^5^), including SMMC-7721, HCC-LY5, MHCC-97L, PLC/PRF/5 and Hep3B cells, were infected with 1×10^6^ recombinant lentivirus-transducing units in the presence of 6 µg/ml polybrene (Sigma-Aldrich, USA).

### Immunohistochemical (IHC) Staining of Human HCC Tissues

Two hundred thirty-six human HCC tissue samples were obtained from patients who underwent surgical treatment at the Guangxi Cancer Institute (Nanning, China), the Qidong Liver Cancer Institute (Qidong, China) or the First Affiliated Hospital of Zhejiang University (Hangzhou, China). The 236 HCC patients included 190 males and 46 females (mean age: 50.9 years, ranging from 21 to 83 years). All procedures were performed under consensus agreements and in accordance with the China Ethical Review Committee. All tissue samples were fixed in 4% phosphate-buffered neutral formalin for at least 72 h and routinely embedded in paraffin. The tissue microarrays were constructed as described previously [22].

Paraffin-embedded tissue array sections (5 µm in thickness) were prepared, and the immunoconjugates were detected by immunofluorescence according to the procedures described previously [11]. For the optimal antibody dilutions, the manufacturers' recommended concentrations were employed. The results were visualized and photographed using an Axioskop 2 microscope (Carl Zeiss, Germany) with a DP70 CCD system (Olympus, Japan).

### Real-time Reverse Transcription-Polymerase Chain Reaction

Total RNA was extracted using TRIzol reagent (Invitrogen, Canada) and reverse transcribed using the PrimeScript™ RT Reagent Kit (Perfect Real Time) (TaKaRa Biotechnology, Japan). The real-time polymerase chain reaction (PCR) was subsequently performed as described previously [22]. The expression levels were normalized against those of the internal reference gene glyceraldehyde-3-phosphate dehydrogenase (GAPDH).

### Western Blot

The cell lysis, sample preparation, SDS-PAGE separation and electrotransferation to nitrocellulose membranes were performed using standard protocols. Immunoblotting was carried out using mouse anti-CD133/1 IgG1 (Miltenyi Biotec) and visualized using SuperSignal West Femto Maximum Sensitivity Substrate (Pierce). β-actin was reprobed as a loading control.

### *In Vivo* Analysis of Tumor Growth and Metastasis

All animal experiment protocols used in this study were approved by the Shanghai Medical Experimental Animal Care Commission at Shanghai Jiaotong University (approval ID. ShCI-12-023). Six- to eight-week-old congenitally immune-deficient nonobese diabetic/severe combined immune-deficiency (NOD/SCID) male mice were randomly divided into groups and maintained under standard conditions according to the institution's guidelines.

For orthotopic inoculation, an 8-mm transverse incision was made in the upper abdomen under anesthesia. Ten thousand CD133^+^ or CD133^−^ cells sorted from SMMC-7721 cells were suspended in 50 µl serum-free DMEM/Matrigel (1∶1) and injected into the left hepatic lobe of the mice using a microsyringe. Tumor formation was monitored starting 1 week after inoculation. The *in vivo* luciferase signal was visualized and measured using an *in vivo* imaging system (LB983 NC320, Berthold Technologies GmbH&Co. KG, Germany). After 12 weeks, all of the mice were sacrificed, and the tumor masses and inoculated murine liver tissue samples were dissected and microscopically examined.

To establish a tumor-homing animal model, NOD/SCID mice were first lavaged with 20 mg/kg 2-acetaminofluorene (2-AAF) or 0.2% DMSO for one week. Next, 2/3 of the left hepatic lobe was surgically resected, and 10,000 CD133^+^ cells or CD133^−^ cells that were freshly isolated from the SMMC-7721 cell line by MACS were injected into the spleen. The spleen was resected 5 minutes after injection, and lavaged with 2-AAF or DMSO continued up to 9 weeks. At the end of the ninth week, all mice were sacrificed, xenograft tumor formation and metastases were observed and the liver and lung tissues were dissected and subjected to microscopic examination [23], [24], [25].

### Statistical analysis

The Statistical Package of Social Sciences software (version 18.0) (SPSS) was used for statistical analysis. The independent Student's t-test or ANOVA was used to compare the continuous variables between the groups, whereas χ2 analysis was applied for comparisons of dichotomous variables. *P* values less than 0.05 were considered statistically significant. Asterisks were used to represent statistical significance of *P* values in some figures, e.g. *p≤0.05, **p≤0.01.

## Results

### CD133^+^ HCC Cells Display High Invasive and Metastatic Potential *In Vitro*

**Figure 1:**
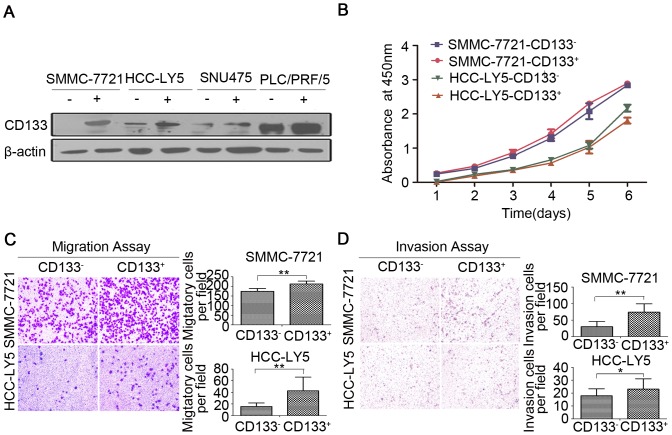
CD133^+^ HCC cells display high invasive and metastatic potential *in vitro*. (A) Isolation of CD133^+^ HCC cells sorted by MACS and detection of CD133 expression in SMMC-7721, HCC-LY5, SNU475, and PLC/PRF/5 cells by Western blot. (B) Growth curves of the sorted CD133^+^ and CD133^−^ SMMC-7721 and HCC-LY5 cells were obtained by the CCK-8 assay. (C) Transwell migration assay of CD133^+^ and CD133^−^ cells sorted from the SMMC-7721 and HCC-LY5 cell lines. (D) Transwell matrigel invasion assay of CD133^+^ and CD133^−^ cells sorted from the SMMC-7721 and HCC-LY5 cell lines.

CSCs are known to exhibit enhanced migratory and invasive potential. To further investigate the metastatic potential of CD133^+^ HCC cells *in vitro*, we sorted SMMC-7721, SNU475, PLC/PRF/5 and primary human HCC-LY5 HCC cells based on CD133 expression by MACS. The CD133 expression of the sorted cells was confirmed by Western blot (Figure 1A), and the CSC-related characteristics of the isolated cells were analyzed. Interestingly, although the CD133^+^ and CD133^−^ cells sorted from the SMMC-7721 and HCC-LY5 cell lines exhibited no significant differences in growth (Figure 1B), the CD133^+^ cells migrated and invaded to a greater extent than the CD133^−^ cells in *in vitro* transwell migration and matrigel invasion assays (Figure 1C, D), indicating that CD133^+^ cells are highly migratory and invasive. To test their proliferative potential, we compared their colony formation abilities by proliferation and soft agar colony formation assays. The results demonstrated that the CD133^+^ cells were able to initiate larger and more numerous colonies than the corresponding CD133^−^ cells (Figure S1, S2), indicating that the CD133^+^ cells exhibit enhanced growth *in vitro*. Beside that, we also tested the self-renewal capacity of sorted CD133 cells by spheroid formation assay. In the first generation, the CD133^+^ cells were able to generate larger and more spheroids than the corresponding CD133^−^ cells *in vitro*. In the second generation, the CD133^+^ cells had maintained their primary character (Figure S3). The results demonstrated that the CD133^+^ cells show active self-renewal capacity *in vitro* in primary and passage cultures.

### CD133^+^ HCC Cells Manifest Highly Metastatic Characteristics *In Vivo*

**Figure 2:**
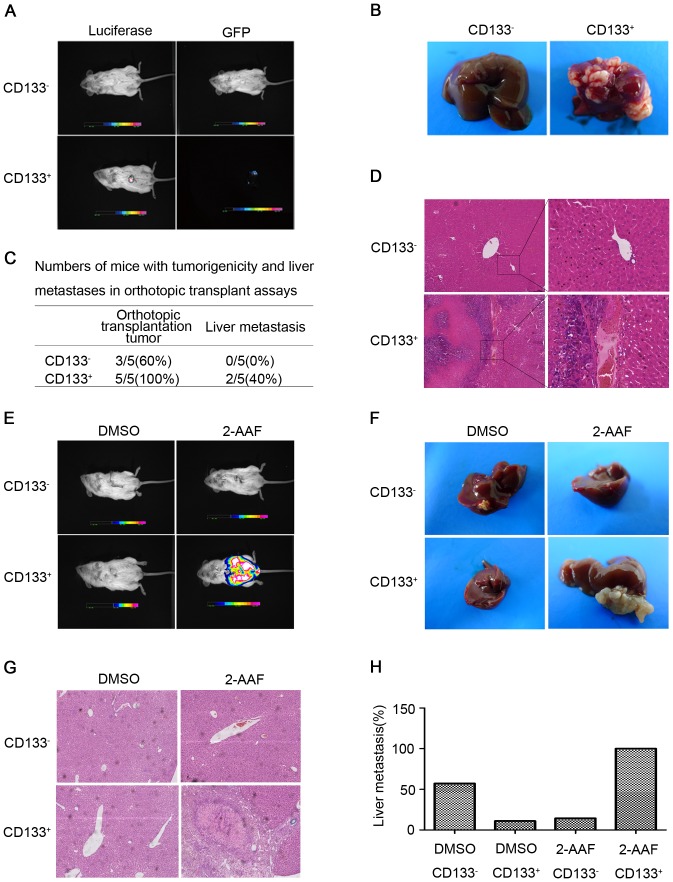
CD133^+^ HCC cells exhibit highly metastatic characteristics *in vivo*. (A) Representative *in vivo* bioluminescence imaging of tumor metastases in NOD/SCID mice after orthotopic transplantation with the isolated CD133^+^ or CD133^−^ SMMC-7721 cells (picture shown is representative of the group orthotopically transplanted with 10,000 cells) (n = 5 each group). (B) Representative examples of NOD/SCID mice orthotopically transplanted with CD133^+^ or CD133^−^ cells isolated from the SMMC-7721 HCC cell line. (C) Numbers of mice manifesting tumorigenicity and liver metastases of CD133^+^ SMMC-7721 cells in the orthotopic transplant assays are shown in the table. (D) HE staining of the harvested tumors confirmed a primary HCC phenotype. (E) Representative *in vivo* bioluminescence imaging of tumor metastasis in the NOD/SCID mice in a tumor-homing animal model transplanted with CD133^+^ or CD133^−^ cells isolated from the SMMC-7721 cell line (n = 9 each group). (F) Representative examples of the liver tumor formation and metastasis in 2-AAF/PHx animal model transplanted with CD133^+^ or CD133^−^ cells isolated from the SMMC-7721 HCC cell line. (G) HE staining of liver tissue sections, a tumor mass from CD133^+^ HCC cells in 2-AAF/PHx animal model was showed. (H) Numbers of mice manifesting liver metastases of CD133^+^ or CD133^−^ SMMC-7721 cells in the tumor-homing animal model are shown in the bar gragh.

Our previous study demonstrated that CD133^+^ HCC cells were distinctly tumorigenic in a xenograft model[11]. To further assess the metastatic potential of the CD133^+^ HCC cells, we established an orthotopic animal transplant model. The results showed that 10,000 CD133^+^ SMMC-7721 cells were sufficient to induce tumor formation in 5/5 (100%) and intrahepatic metastasis in 2/5 (40%) NOD/SCID mice after 3 months, whereas an equivalent number of CD133^−^ cells only induced tumor formation in 3/5 (60%) mice and did not induce tumor metastasis (Figure 2A, B, C). Hematoxylin-eosin (HE) staining revealed similar histological characteristics in the orthotopic tumor transplants (Figure 2D). In addition, to further assess the *in vivo* tumor cell homing capacity, which is considered a metastatic characteristic of CSCs, an animal model of tumor cell homing was established in NOD/SCID mice. The results showed that 9/9 NOD/SCID mice inoculated with 10,000 CD133^+^ HCC cells developed tumors and metastasis, whereas fewer tumors were found in the livers of NOD/SCID mice treated with an equivalent number of CD133^−^ cells. Interestingly, the tumors generated by the CD133^+^ cells grew and formed tumor mass at the site of the resected liver lobe, indicating that the CD133^+^ HCC cells are highly mobile and exhibit a tumor-homing capacity (Figure 2E, F), which were confirmed by histopathological examination (Figure 2G, H). The results presented above firmly indicated that CD133^+^ cells possessed a high capacity for tumor metastasis *in vivo* in HCC.

### CD133^+^ HCC Cells Display Unique Gene Expression Profiles

**Figure 3:**
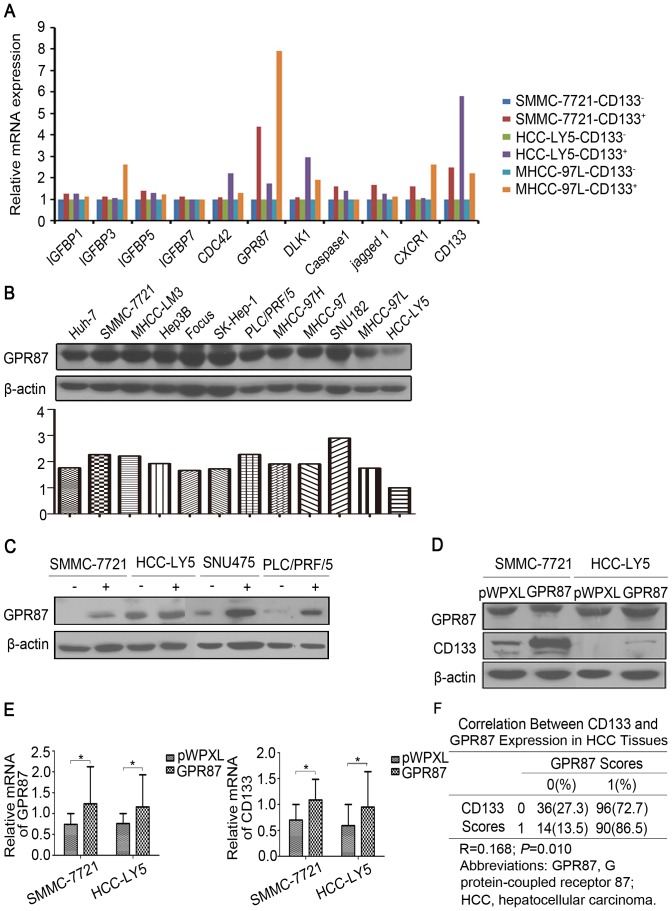
Detection of GPR87 expression in HCC cell lines and human primary cells by qRT-PCR and Western blot analysis. (A) Relative mRNA expression levels of GPR87 and CD133 were determined by quantitative polymerase chain reaction in the CD133^+^ and CD133^−^ populations sorted from the SMMC-7721, HCC-LY5 and MHCC-97L cell lines. (B) Western blot of GPR87 in the HCC cell lines and primary human cells. (C) Western blot of GPR87 in the CD133^+^ and CD133^−^ populations sorted from the SMMC-7721, HCC-LY5, SNU475 and PLC/RFP/5 cells. (D) The protein expression levels of GPR87 and CD133 were determined by Western blot analysis in the SMMC-7721-lenti-GPR87 and HCC-LY5-lenti-GPR87 cells. (E) The relative mRNA expression of GPR87 and CD133 were determined by quantitative polymerase chain reaction in the SMMC-7721-lenti-GPR87 and HCC-LY5-lenti-GPR87 cells. (F) The table showed the correlation between CD133 and GPR87 expression in HCC tissues.

To study the molecular mechanism underlying metastasis in human hepatocellular carcinoma, we compared the global gene expression profiles of the CD133^+^ HCC cells and their CD133^−^ counterparts isolated from SMMC-7721 cells using the Affymetrix GeneChip® Human Genome U133 Plus 2.0 Array. We identified 454 common differentially expressed genes that displayed a fold change of greater than 1.5. Of these 454 genes, 312 were up-regulated and 142 were down-regulated. The identified genes were further subjected to bioinformatic analysis. The functions of the differentially expressed genes were associated with cell adhesion, migration, cytoskeleton, chemotactic movement and metastasis (Table S1, S2). Among the genes that were differentially expressed between the CD133^+^ and CD133^−^ populations, GPR87 was found to be up-regulated by approximately 8-fold in the CD133^+^ cells compared with the CD133^−^ cells. To clarify the role of GPR87 in the tumor growth and metastases of CD133^+^ HCC cells, we first examined the expression of GPR87 in sorted HCC cell lines and primary human HCC cells by qRT-PCR and Western blot. We found that the expression levels of GPR87 in the CD133^+^ HCC cell lines were higher than in their CD133^−^ counterparts (Figure 3A, C). We then tested the capacity of different HCC cell lines and human primary cells to express GPR87. GPR87 was detected in the HCC cell lines and primary human HCC cells by Western blot analysis, although in different amounts (Figure 3B). To better understand the function of GPR87 in HCC metastasis, we first established two stable cell lines ectopically expressing GPR87, SMMC-7721-lenti-GPR87 and HCC-LY5-lenti-GPR87, using a lentivirus vector. We then examined CD133 expression and CSC-related properties in these stable cell lines. We found that the overexpression of GPR87 in SMMC-7721 and HCC-LY5 cells resulted in an increase in the mRNA and protein levels of CD133 (Figure 3D, E). To better understand the correlation between CD133 and GPR87 expression in HCC tissues, immunohistochemical staining was performed. CD133 and GPR87 expression was detected in tumor specimens, the expression of CD133 was correlated with the expression of GPR87 in HCC tissues (R = 0.168, P<0.05) (Figure 3F; Table S3).

### Overexpression of GPR87 Up-regulates CD133 Expression and CSC-related Properties

**Figure 4:**
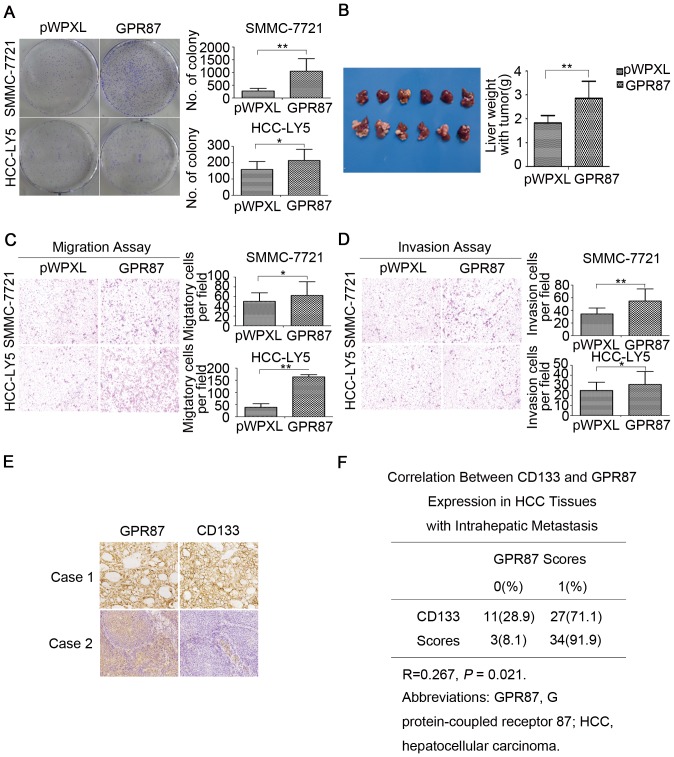
Overexpression of GPR87 up-regulates CD133 expression and enhances CSC-related properties. (A) Representative examples of proliferation assays examining the effect of GPR87 overexpression in SMMC-7721 and HCC-LY5 cells. (B) The gross features of the tumor-bearing NOD/SCID mice orthotopically transplanted with 2×10^6^ SMMC-7721-lenti-GPR87 and SMMC-7721-lenti- pWPXL cells after 6 weeks (n = 6 each group). (C) Transwell migration assay in SMMC-7721 and HCC-LY5 cells overexpressing GPR87. (D) Transwell matrigel invasion assay in SMMC-7721 and HCC-LY5 cells overexpressing GPR87. (E) Immunohistochemical staining of GPR87 and CD133 in HCC tissues with intrahepatic metastasis. (F) The table showed the correlation between CD133 and GPR87 expression in HCC tissues with intrahepatic metastasis.

To determine the effect of GPR87 on cell proliferation, we analyzed the role of GPR87 in colony formation. As depicted in Figure 4A, the overexpression of GPR87 in SMMC-7721 and HCC-LY5 cells increased the colony formation capacity compared with the corresponding empty vector-infected cells (P<0.05). To examine whether the overexpression of GPR87 could also promote tumor growth and metastasis *in vivo*, we orthotopically inoculated 2×10^6^ SMMC-7721-lenti-GPR87 or SMMC-7721-lenti-control cells into the left hepatic lobe of NOD/SCID mice with a microsyringe. Gross examination revealed that 6/6 mice orthotopically inoculated with SMMC-7721-lenti-GPR87 cells developed tumors after 6 weeks, whereas tumor development was only detected in 3/6 mice treated with an equivalent number of SMMC-7721-lenti-control cells (Figure 4B). In addition, the average tumor size in the mice inoculated with SMMC-7721-lenti-GPR87 cells was 1.6-fold larger than that in mice inoculated with SMMC-7721-lenti-pWPXL cells. *In vitro* transwell migration and matrigel invasion assays showed that SMMC-7721-lenti-GPR87 and HCC-LY5-lenti-GPR87 cells migrated and invaded to a greater extent than the empty vector-infected cells (P<0.05) (Figure 4C, D). These findings indicate that GPR87 can up-regulate CD133 expression and promote metastasis *in vitro* and growth *in vivo*. Although CD133 protein levels in HCC tissues without intrahepatic metastasis were not correlated with GPR87 expression (R = 0.120, P>0.05) (Table S4, S5), the CD133 protein levels in HCC tissues with intrahepatic metastasis positively correlated with GPR87 expression (R = 0.267, P<0.05) (Figure 4E, F; Table 1), suggesting that CD133 might be up-regulated by the expression of GPR87 in metastatic HCC.

**Table 1:** Correlation Between CD133 and GPR87 Expression Levels in HCC Patients with Intrahepatic Metastasis and Their Clinicopathologic Characteristics.

Clinical Pathology		CD133			GPR87		
		Negative(%)	Positive(%)	*P* Value	Negative(%)	Positive(%)	*P* Value
Gender	Male	29 (50.9)	28(49.1)	0.948	10(17.5)	47(82.5)	0.657
	Female	9(50.0)	9(50.0)		4(22.2)	14(77.8)	
Age	≤50	28(51.9)	26(48.1)	0.742	11(20.4)	43(79.6)	0.544
	>50	10(47.6)	11(52.4)		3(14.3)	18(85.7)	
AFP (ng/mL)	≤20	7(33.3)	14(66.7)	0.083	3(14.3)	18(85.7)	0.500
	>20	29(55.8)	23(44.2)		11(21.2)	41(78.8)	
HBsAg	Absent	4(33.3)	8(66.7)	0.170	2(16.7)	10(83.3)	0.790
	Present	33(55.0)	27(45.0)		12(20.0)	48(80.0)	
antiHBs	Absent	34(51.5)	32(48.5)	0.620	13(19.7)	53(80.3)	0.987
	Present	2(40.0)	3(60.0)		1(20.0)	4(80.0)	
HBeAg	Absent	29(49.2)	30(50.8)	0.378	12(20.3)	47(79.7)	0.870
	Present	7(63.6)	4(36.4)		2(18.2)	9(81.8)	
antiHBe	Absent	20(50.0)	20(50.0)	1.000	10(25.0)	30(75.0)	0.183
	Present	16(50.0)	16(50.0)		4(12.5)	28(87.5)	
antiHBc	Absent	5(33.3)	10(66.7)	0.130	3(20.0)	12(80.0)	0.975
	Present	31(55.4)	25(44.6)		11(19.6)	45(80.4)	
antiHCV	Absent	12(46.2)	14(53.8)	0.849	5(19.2)	21(80.8)	0.179
	Present	4(50.0)	4(50.0)		0(0.0)	8(100.0)	
Histological grade	I–II	8(53.3)	7(46.7)	0.817	2(13.3)	13(86.7)	0.553
	III–IV	30(50.0)	30(50.0)		12(20.0)	48(80.0)	
Tumor size (cm)	≤5	17(44.7)	21(55.3)	0.222	6(15.8)	32(84.2)	0.514
	>5	19(59.4)	13(40.6)		7(21.9)	25(78.1)	
Cirrhosis	Absent	0(0.0)	1(100.0)	0.308	0(0.0)	1(100.0)	0.630
	Present	38(51.4)	36(48.6)		14(18.9)	60(81.1)	

*P* value represents the probability from a chi-square test for CD133 and GPR87 expression levels between variable subgroups.

**Abbreviations**: GPR87, G protein-coupled receptor 87; HCC, hepatocellular carcinoma; AFP, alpha-fetoprotein; HBsAg, hepatitis B surface antigen; antiHBs, anti-hepatitis B surface antibody; HBeAg, hepatitis B e antigen; antiHBe, anti-hepatitis B e antibody; antiHBc, anti-hepatitis B core antibody; antiHCV, anti-hepatitis C virus antibody.

### Silencing of GPR87 Inhibits CD133 Expression and CSC-related Properties

**Figure 5:**
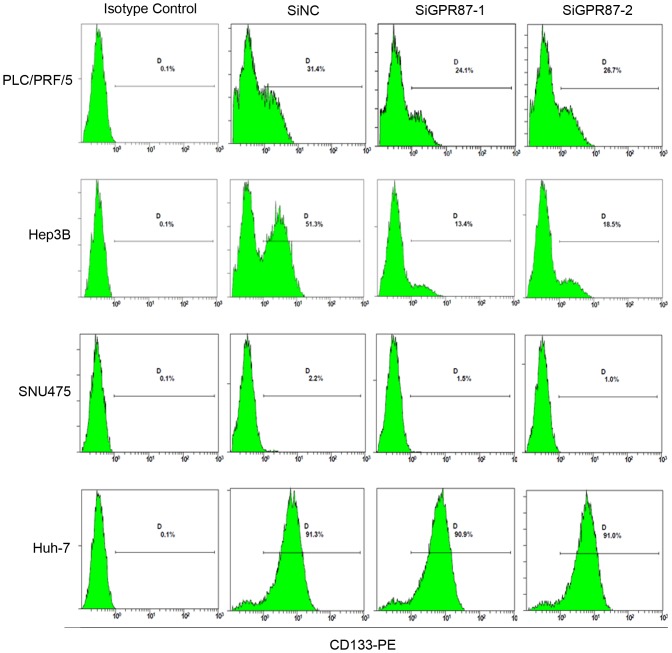
Silencing of GPR87 reduces the levels of CD133 expression. Flow cytometric analysis of the levels of CD133 expression in GPR87 siRNA-treated cells, including the PLC/PRF/5, Hep3B, SNU475 and Huh-7 cell lines.

To investigate the functional role of GPR87 in CD133^+^ HCC cells, we inhibited the expression of GPR87 in PLC/PRF/5, Hep3B, SNU475 and Huh-7 cells using siRNA transfection. Flow cytometric analysis revealed that the silencing of GPR87 reduced the levels of CD133^+^ cells expression from 31.4% to 24.1% and 26.7% in PLC/PRF/5 cells, from 51.3% to 13.4% and 18.5% in Hep3B cells, from 2.2% to 1.5% and 1.0% in SNU475 cells, but no change was detected in the Huh-7 cells (Figure 5). *In vitro*, the silencing of GPR87 in PLC/PRF/5 and Hep3B cells resulted in a decrease in the mRNA and protein expression of CD133 (Figure S4). Moreover, GPR87 shRNA resulted in reduced cell growth (P<0.05) (Figure S5). We also tested the ability of cells to invade, migrate capacity following GPR87 knockdown. *In vitro* transwell migration and matrigel invasion assays showed that there was a decrease in PLC/PRF/5-shGPR87 cells than the empty vector-infected cells (P<0.05) (Figure S6). These data suggest that GPR87 plays a major role in regulating CD133 expression.

### GPR87 Mediates the Expression of CD133 in HCC Cell Lines

**Figure 6:**
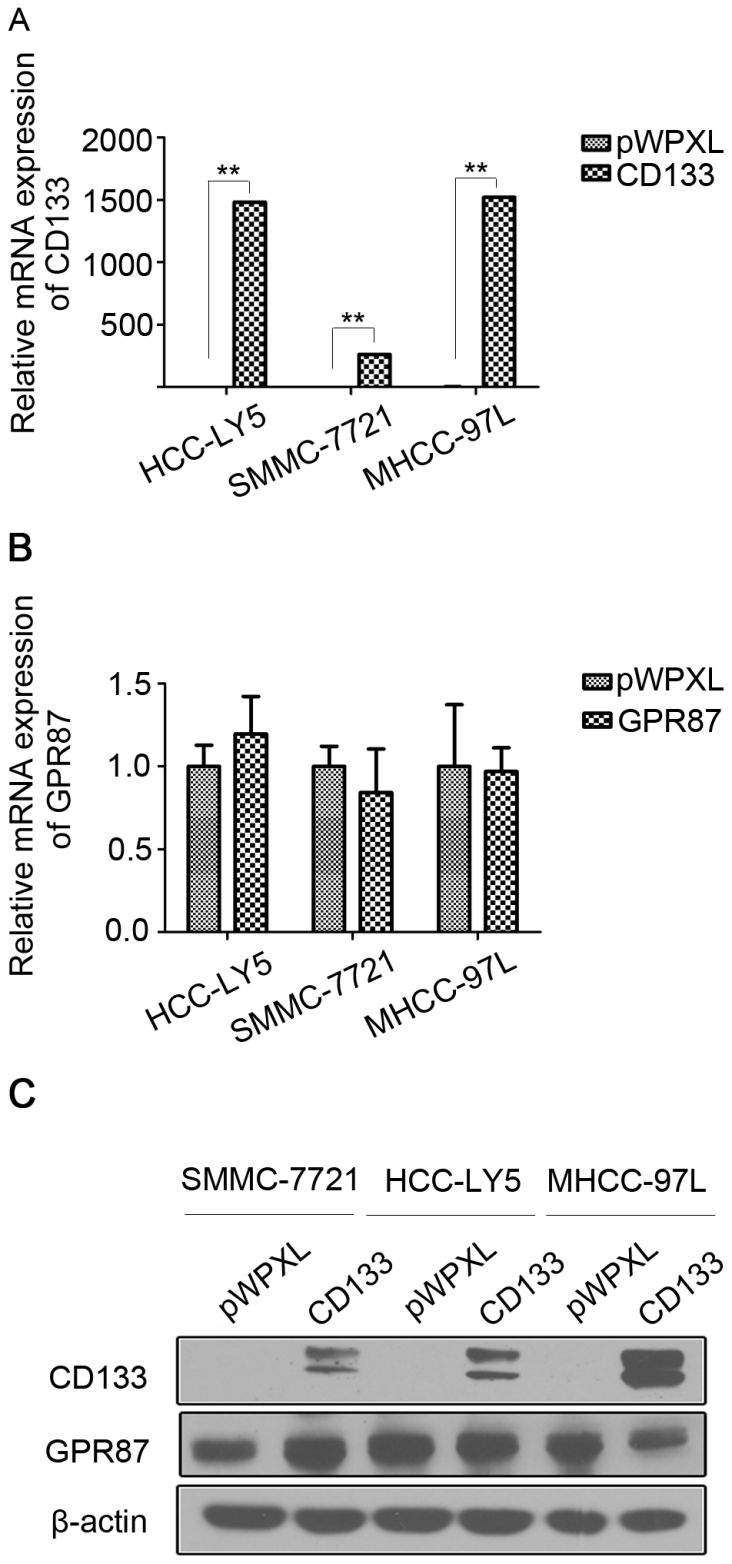
GPR87 mediates the expression of CD133 in HCC cell lines. (A) Relative mRNA expression of CD133 in SMMC-7721-lenti-CD133, HCC-LY5-lenti-CD133 and MHCC-97L-lenti-CD133 cells. (B) Relative mRNA expression of GPR87 in SMMC-7721-lenti-CD133, HCC-LY5-lenti-CD133 and MHCC-97L-lenti-CD133 cells. (C) Western blot of GPR87 and CD133 in SMMC-7721-lenti-CD133, HCC-LY5-lenti-CD133 and MHCC-97L-lenti-CD133 cells.

As experiments shown above, we had proved that knockdown of GPR87 could inhibit the expression of CD133 and *in vitro* spreading, while overexpression of GPR87 could promote the expression of CD133 and increase tumor metastasis in HCC. To further explore the correlation of GPR87 and CD133 expression, we established stable CD133 overexpressing cell lines, SMMC-7721-lenti-CD133, HCC-LY5-lenti-CD133 and MHCC-97L-lenti-CD133, using a lentivirus vector, then examined GPR87 expression. The results clearly showed that overexpression of CD133 were not able to up-regulate the expression of GPR87 by qRT-PCR (Figure 6A, B) and western blotting (Figure 6C).

## Discussion

Cancer is a systemic disease, and metastasis accounts for over 90% of lethality in cancer patients [26]. In HCC, metastasis is one of the most valuable prognostic factors and significantly affects patient outcomes [27]. Recently, cancer stem cells (CSCs) have been identified as a subpopulation of cancer cells that are responsible for tumorigenesis, therapeutic resistance, recurrence and metastasis [28], [29], [30], [31]. However, the molecular mechanism of CSC metastasis remains unclear. Further exploring this mechanism may not only provide new insight into HCC but also identify a useful molecular target for efficiently treating HCC.

Recently, we and others have identified a CSC population in hepatocellular carcinoma characterized by the expression of CD133. However, the detailed metastatic characteristics of HCC cells expressing CD133 remain unclear. In this study, we show that the CD133^+^ HCC cells possess high invasive and metastatic potential *in vitro*. Unlike their CD133^−^ counterparts, 10,000 CD133^+^ SMMC-7721 cells were sufficient to induce orthotopic tumor formation and intrahepatic metastasis in NOD/SCID mice after 3 months. In addition, we have demonstrated that 10,000 CD133^+^ cells were sufficient to induce experimental metastasis upon spleenic inoculation in a tumor-homing animal model, indicating that CD133^+^ cells have tumor-homing capacity *in vivo*.

Recently, the increased expression of CD133 has been observed in the cancer stem cells of a variety of human and mouse cancers. Emerging evidence has demonstrated that CD133 expression can be regulated by multiple factors, including transforming growth factor beta 1[32], BMP4 [33], microRNA-150 [2] and Interferon-alpha [4]. However, no natural ligand for CD133 has yet been identified, and little is known about its function. In the present study, we compared the global gene expression profiles of CD133^+^ HCC CSCs and their CD133^−^ counterparts isolated from SMMC-7721 cells using GeneChip analysis and found that the expression of GPR87 in CD133^+^ HCC cell lines was increased compared to that in their CD133^−^ counterparts. This finding indicates that there may be a link between GPR87 and CD133 in the process of metastasis in HCC. However, no evidence has confirmed the correlation between GPR87 and CD133. Here, we demonstrate for the first time that the silencing of GPR87 decreased the CD133 expression levels. The overexpression of GPR87 significantly enhanced the migration and invasion of HCC cells, increased their colony formation capacity *in vitro* and promoted tumor formation and growth *in vivo*. These results suggest that GPR87 has a critical role in modulating the expression of CD133 and contributes to the growth and metastasis of HCC cells.

The molecular mechanism underlying metastasis in HCC CSCs requires further study. Our future work will focus on elucidating the mechanism underlying the GPR87-mediated regulation of CD133 in HCC CSCs and defining molecular therapeutic targets on HCC CSCs.

## Supporting Information

Figure S1 **Colony formation assay of CD133^+/−^ HCC cells at 2D culture.** Representative examples of proliferation assays of CD133^+^ and CD133^−^ cells isolated from SMMC-7721 and HCC-LY5 cells.
(TIF)

Figure S2 **Colony formation assay of CD133^+/−^ HCC cells in soft agar.** Representative examples of proliferation assays of CD133^+^ and CD133^−^ cells isolated from SMMC-7721 and HCC-LY5 cells.
(TIF)

Figure S3 **Spheroid formation assay of CD133^+/−^ HCC cells** ***in vitro*** **.** Representative examples of spheroid formation assays of CD133^+^ and CD133^−^ cells isolated from SMMC-7721 and HCC-LY5 cells.
(TIF)

Figure S4 **Quality control of established stable lenti-shGPR87 expressing HCC cell lines.** Relative mRNA expression of GPR87 and CD133 were determined by quantitative polymerase chain reaction in the silencing of GPR87 PLC/PRF/5 and Hep3B cells.
(TIF)

Figure S5 **Growth curve of stable lenti-shGPR87 expressing HCC cell lines.** Growth curves of stable knockdown of GPR87 in PLC/PRF/5 and Hep3B cells were obtained by CCK-8 assay.
(TIF)

Figure S6 ***In vitro*** **migration and invasion assays of stable knockdown of GPR87 in PLC/PRF/5 cell line.** Representative examples of transwell migration and matrigel invasion assay in PLC/PRF/5 knockdown GPR87.
(TIF)

Table S1 **Selected up-regulated genes.**
(DOC)

Table S2 **Selected down-regulated genes.**
(DOC)

Table S3 **Correlation Between CD133 and GPR87 Expression Levels in HCC Patients and Their Clinicopathologic Characteristics.**
(DOC)

Table S4 **Correlation Between CD133 and GPR87 Expression Levels in HCC Patients without Intrahepatic Metastasis and Their Clinicopathologic Characteristics.**
(DOC)

Table S5 **Correlation Between CD133 and GPR87 Expression in HCC Tissues without Intrahepatic Metastasis.**
(DOC)
