# Supplementary figures and images for: G Protein-Coupled Receptor 87 (GPR87) Promotes the Growth and Metastasis of CD133+ Cancer Stem-Like Cells in Hepatocellular Carcinoma
Source: PLoS One. 2013 Apr 10;8(4):e61056. doi: 10.1371/journal.pone.0061056 (PMC3622685; doi:10.1371/journal.pone.0061056)

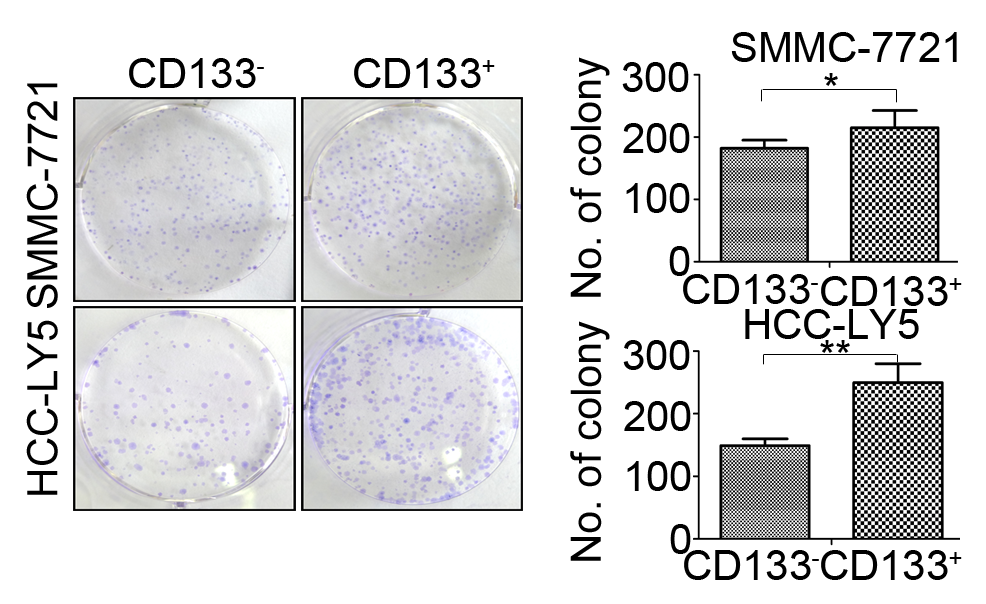

Supplement: Figure S1 — Colony formation assay of CD133+/− HCC cells at 2D culture. Representative examples of proliferation assays of CD133+ and CD133− cells isolated from SMMC-7721 and HCC-LY5 cells. (TIF) [file pone.0061056.s001.tif]

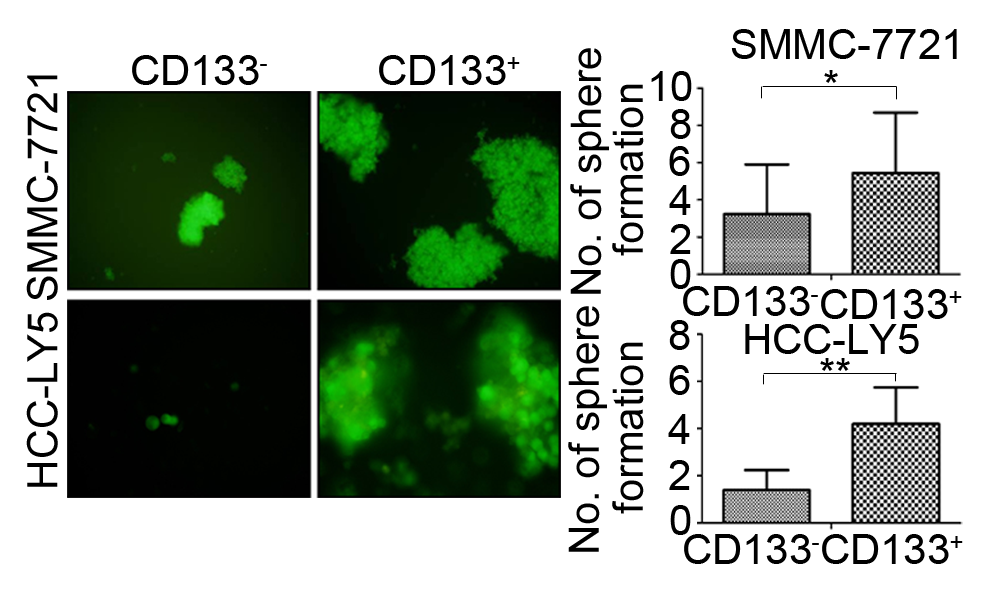

Supplement: Figure S2 — Colony formation assay of CD133+/− HCC cells in soft agar. Representative examples of proliferation assays of CD133+ and CD133− cells isolated from SMMC-7721 and HCC-LY5 cells. (TIF) [file pone.0061056.s002.tif]

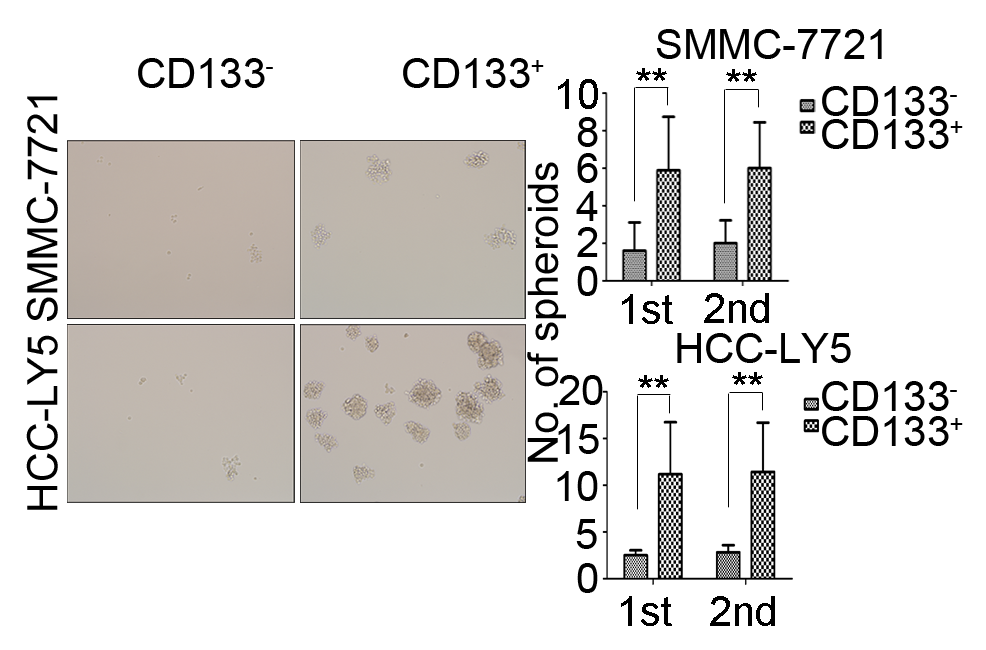

Supplement: Figure S3 — Spheroid formation assay of CD133+/− HCC cells in vitro . Representative examples of spheroid formation assays of CD133+ and CD133− cells isolated from SMMC-7721 and HCC-LY5 cells. (TIF) [file pone.0061056.s003.tif]

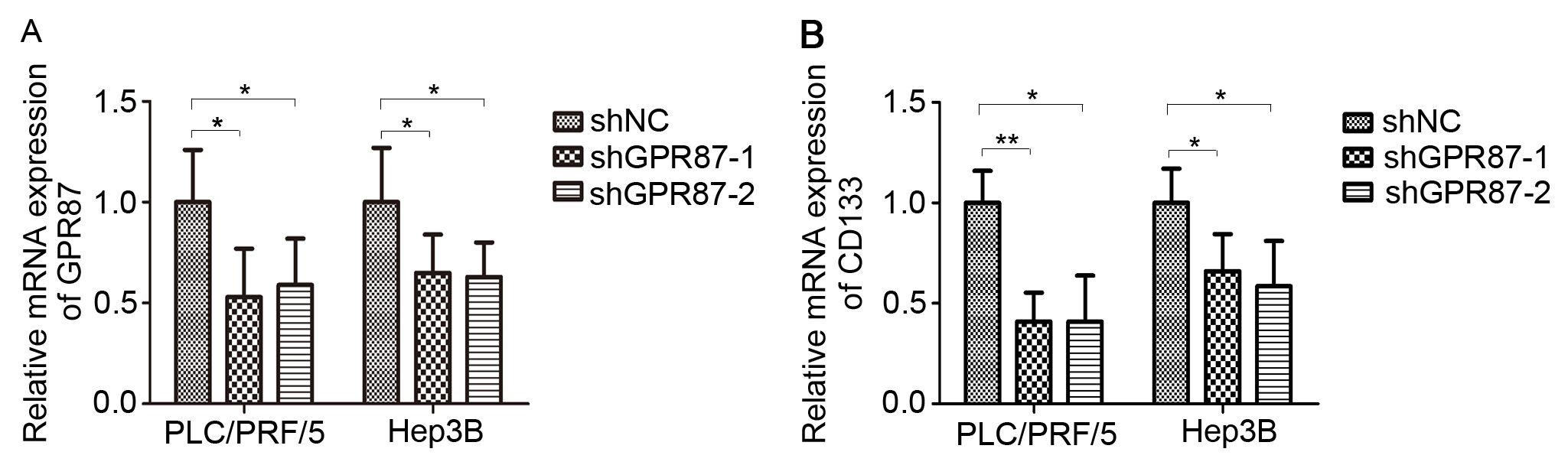

Supplement: Figure S4 — Quality control of established stable lenti-shGPR87 expressing HCC cell lines. Relative mRNA expression of GPR87 and CD133 were determined by quantitative polymerase chain reaction in the silencing of GPR87 PLC/PRF/5 and Hep3B cells. (TIF) [file pone.0061056.s004.tif]

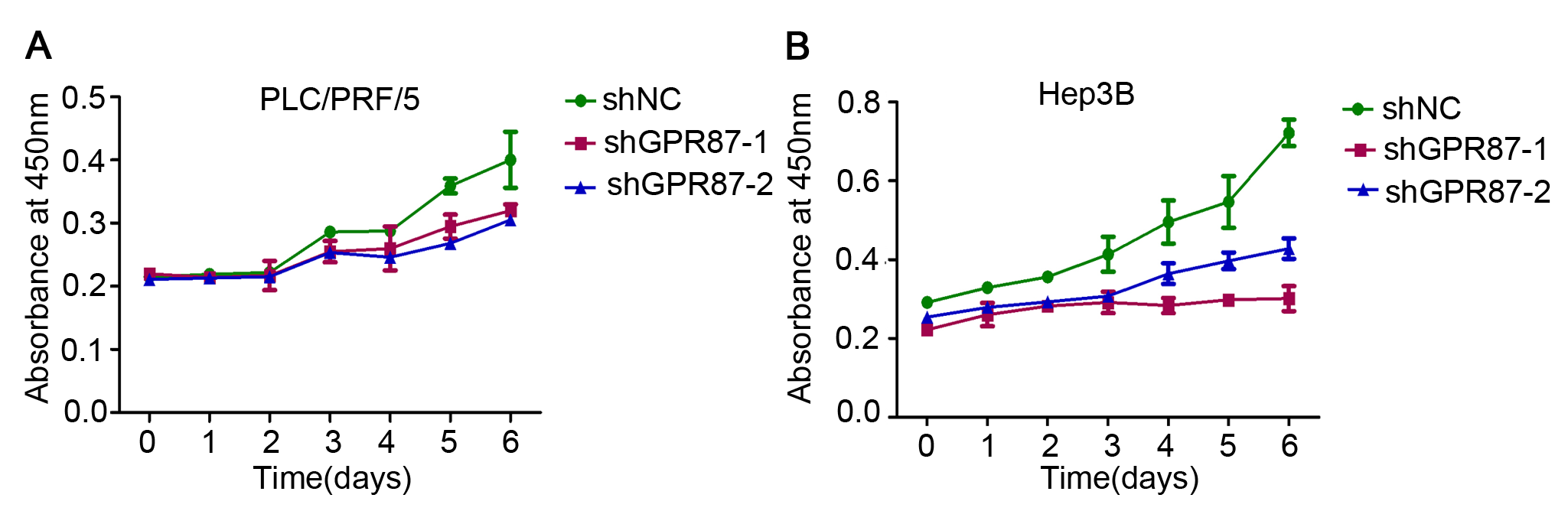

Supplement: Figure S5 — Growth curve of stable lenti-shGPR87 expressing HCC cell lines. Growth curves of stable knockdown of GPR87 in PLC/PRF/5 and Hep3B cells were obtained by CCK-8 assay. (TIF) [file pone.0061056.s005.tif]

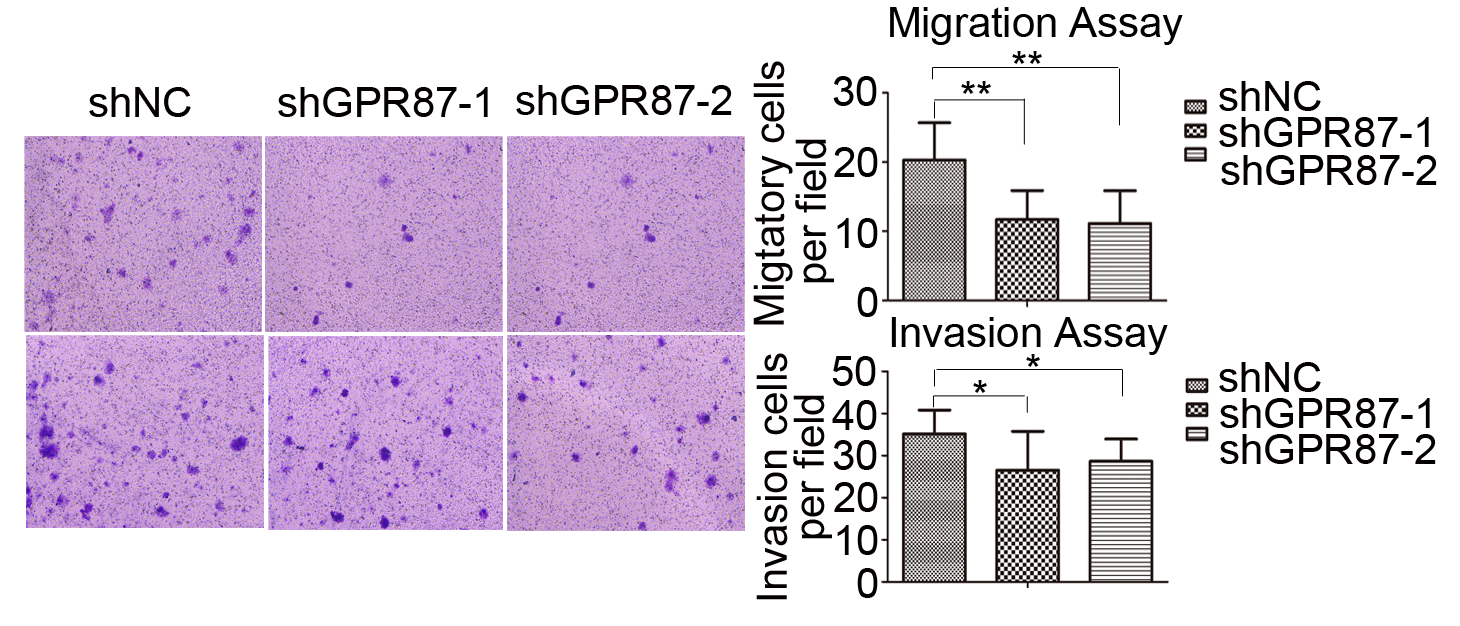

Supplement: Figure S6 — In vitro migration and invasion assays of stable knockdown of GPR87 in PLC/PRF/5 cell line. Representative examples of transwell migration and matrigel invasion assay in PLC/PRF/5 knockdown GPR87. (TIF) [file pone.0061056.s006.tif]
