# Supplementary material for: G Protein-Coupled Receptor 87 (GPR87) Promotes the Growth and Metastasis of CD133+ Cancer Stem-Like Cells in Hepatocellular Carcinoma
Source: PLoS One. 2013 Apr 10;8(4):e61056. doi: 10.1371/journal.pone.0061056 (PMC3622685; doi:10.1371/journal.pone.0061056)
Supplement: Table S1 — Selected up-regulated genes. (DOC) [file pone.0061056.s007.doc]

**Table S**1. Selected up-regulated genes.

| Probe Set ID | Gene Symbol | Gene Title | Ratio |
| --- | --- | --- | --- |
| 211367_s_at | CASP1 | caspase 1, apoptosis-related cysteine peptidase (interleukin 1, beta, convertase) | 11.51 |
| 219936_s_at | GPR87 | G protein-coupled receptor 87 | 7.533 |
| 209735_at | ABCG2 | ATP-binding cassette sub-family G  member 2 | 7.427 |
| 218795_at | ACP6 | acid phosphatase 6, lysophosphatidic | 7.355 |
| 221729_at | COL5A2 | collagen, type V, alpha 2 | 7.307 |
| 218718_at | PDGFC | platelet derived growth factor C | 7.233 |
| 232449_at | BCO2 | beta-carotene oxygenase 2 | 6.598 |
| 205603_s_at | DIAPH2 | diaphanous homolog 2 (Drosophila) | 6.555 |
| 204137_at | GPR137B | G protein-coupled receptor 137B | 6.527 |
| 209699_x_at | AKR1C2 | dehydrogenase, type III) | 6.504 |
| 220393_at | LGSN | lengsin, lens protein with glutamine synthetase domain | 6.465 |
| 214954_at | SUSD5 | sushi domain containing 5 | 6.212 |
| 203434_s_at | MME | membrane metallo-endopeptidase | 6.12 |
| 204359_at | FLRT2 | fibronectin leucine rich transmembrane protein 2 | 5.914 |
| 220327_at | VGLL3 | vestigial like 3 (Drosophila) | 5.759 |
| 221577_x_at | GDF15 | growth differentiation factor 15 | 5.543 |
| 242414_at | QPRT | quinolinate phosphoribosyltransferase | 5.233 |
| 244552_at | ZNF788 | zinc finger family member 788 | 5.185 |
| 204431_at | TLE2 | transducin-like enhancer of split 2 (E(sp1) homolog | 5.119 |
| 225355_at | NEURL1B | neuralized homolog 1B (Drosophila) | 4.961 |
