# Supplementary material for: G Protein-Coupled Receptor 87 (GPR87) Promotes the Growth and Metastasis of CD133+ Cancer Stem-Like Cells in Hepatocellular Carcinoma
Source: PLoS One. 2013 Apr 10;8(4):e61056. doi: 10.1371/journal.pone.0061056 (PMC3622685; doi:10.1371/journal.pone.0061056)
Supplement: Table S2 — Selected down-regulated genes. (DOC) [file pone.0061056.s008.doc]

**Table S**2. Selected down-regulated genes.

| Probe Set ID | Gene Symbol | Gene Title | Ratio |
| --- | --- | --- | --- |
| 207935_s_at | KRT13 | keratin 13 | 0.052 |
| 202465_at | PCOLCE | procollagen C-endopeptidase enhancer | 0.123 |
| 1563638_at | FAM18A | family with sequence similarity 18,  member A | 0.13 |
| 213240_s_at | KRT4 | keratin 4 | 0.149 |
| 202575_at | CRABP2 | cellular retinoic acid binding protein 2 | 0.194 |
| 208025_s_at | HMGA2 | high mobility group AT-hook 2 | 0.307 |
| 219305_x_at | FBXO2 | F-box protein 2 | 0.321 |
| 238029_s_at | SLC16A14 | solute carrier family 16, member 14 | 0.323 |
| 223549_s_at | ESPN | espin | 0.324 |
| 201288_at | ARHGDIB | Rho GDP dissociation inhibitor (GDI) beta | 0.332 |
| 214586_at | GPR37 | G protein-coupled receptor 37 | 0.356 |
| 224997_x_at | H19 | H19, imprinted maternally expressed transcript | 0.359 |
| 222073_at | COL4A3 | collagen, type IV, alpha 3 (Goodpasture antigen) | 0.36 |
| 204288_s_at | SORBS2 | sorbin and SH3 domain containing 2 | 0.362 |
| 219370_at | RPRM | reprimo, TP53 dependent G2 arrest mediator candidate | 0.363 |
| 211748_x_at | PTGDS | prostaglandin D2 synthase 21kDa (brain) | 0.37 |
| 1565799_at | RAB3IP | RAB3A interacting protein (rabin3) | 0.379 |
| 215076_s_at | COL3A1 | collagen, type III, alpha 1 | 0.382 |
| 238615_at | ERLIN2 | ER lipid raft associated 2 | 0.39 |
| 212531_at | LCN2 | lipocalin 2 | 0.391 |
