# Supplementary material for: G Protein-Coupled Receptor 87 (GPR87) Promotes the Growth and Metastasis of CD133+ Cancer Stem-Like Cells in Hepatocellular Carcinoma
Source: PLoS One. 2013 Apr 10;8(4):e61056. doi: 10.1371/journal.pone.0061056 (PMC3622685; doi:10.1371/journal.pone.0061056)
Supplement: Table S3 — Correlation Between CD133 and GPR87 Expression Levels in HCC Patients and Their Clinicopathologic Characteristics. (DOC) [file pone.0061056.s009.doc]

**Table S**3. Correlation Between CD133 and GPR87 Expression Levels in HCC Patients and Their Clinicopathologic Characteristics.

| Clinical Pathology | | CD133 | | | GPR87 | | |
| --- | --- | --- | --- | --- | --- | --- | --- |
|  |  | Negative  (%) | Positive  (%) | *P* Value | Negative  (%) | Positive  (%) | *P* Value |
| Gender | Male | 104(54.7) | 86(45.3) | 0.452 | 36(18.9) | 154(81.1) | 0.087 |
|  | Female | 28(60.9) | 18(39.1) |  | 14(30.4) | 32(69.6) |  |
| Age | ≤50 | 88(55.3) | 71(44.6) | 0.713 | 41(25.8) | 118(74.2) | 0.015^*^ |
|  | >50 | 44(57.9) | 32(42.1) |  | 9(11.8) | 67(88.2) |  |
| AFP (ng/mL) | ≤20 | 39(49.4) | 40(50.6) | 0.201 | 17(21.5) | 62(78.5) | 0.823 |
|  | >20 | 89(58.2) | 64(41.8) |  | 31(20.3) | 122(79.7) |  |
| HBsAg | Absent | 21(50.0) | 21(50.0) | 0.394 | 10(23.8) | 32(76.2) | 0.673 |
|  | Present | 107(57.2) | 80(26.7) |  | 39(20.9) | 148(79.1) |  |
| HBeAg | Absent | 96(52.5) | 87(47.5) | 0.072 | 40(21.9) | 143(78.1) | 0.805 |
|  | Present | 26(68.4) | 12(31.6) |  | 9(23.7) | 29(76.3) |  |
| antiHBe | Absent | 77(63.1) | 45(36.9) | 0.007^*^ | 30(24.6) | 92(75.4) | 0.318 |
|  | Present | 45(45.0) | 55(55.0) |  | 19(19.0) | 81(81.0) |  |
| antiHBc | Absent | 28(57.1) | 21(42.9) | 0.757 | 9(18.4) | 40(81.6) | 0.467 |
|  | Present | 94(54.7) | 78(45.3) |  | 40(23.2) | 132(76.7) |  |
| antiHCV | Absent | 49(49.0) | 51(51.0) | 0.742 | 26(26.0) | 74(74.0) | 0.036^*^ |
|  | Present | 7(53.8) | 6(46.2) |  | 0(0.0) | 13(100.0) |  |
| Histological grade | Ⅰ-Ⅱ | 70(58.8) | 49(41.2) | 0.367 | 25(21.0) | 94(79.0) | 0.946 |
|  | Ⅲ-Ⅳ | 62(53.0) | 55(47.0) |  | 25(21.4) | 92(78.6) |  |
| Tumor size (cm) | ≤5 | 63(55.8) | 50(44.2) | 0.759 | 22(19.5) | 91(80.5) | 0.696 |
|  | >5 | 67(57.8) | 49(42.2) |  | 25(21.6) | 91(78.4) |  |
| Cirrhosis | Absent | 20(18.9) | 18(47.4) | 0.655 | 8(21.1) | 30(78.9) | 0.982 |
|  | Present | 112(56.6) | 86(43.9) |  | 42(21.4) | 156(79.6) |  |
| Intrahepatic metastasis | Absent | 94(58.4) | 67(41.6) | 0.266 | 36(22.4) | 125(77.6) | 0.518 |
|  | Present | 38(50.7) | 37(49.3) |  | 14(18.7) | 61(81.3) |  |

*P* value represents the probability from a chi-square test for CD133 and GPR87 expression

levels between variable subgroups.

**P* < 0.05.

**Abbreviations:** GPR87, G protein-coupled receptor 87; HCC, hepatocellular carcinoma;

AFP, alpha-fetoprotein; HBsAg, hepatitis B surface antigen; antiHBs, anti-hepatitis B surface antibody; HBeAg, hepatitis B e antigen; antiHBe, anti-hepatitis B e antibody; antiHBc, anti-hepatitis B core antibody; antiHCV, anti-hepatitis C virus antibody.
