# Supplementary material for: G Protein-Coupled Receptor 87 (GPR87) Promotes the Growth and Metastasis of CD133+ Cancer Stem-Like Cells in Hepatocellular Carcinoma
Source: PLoS One. 2013 Apr 10;8(4):e61056. doi: 10.1371/journal.pone.0061056 (PMC3622685; doi:10.1371/journal.pone.0061056)
Supplement: Table S4 — Correlation Between CD133 and GPR87 Expression Levels in HCC Patients without Intrahepatic Metastasis and Their Clinicopathologic Characteristics. (DOC) [file pone.0061056.s010.doc]

**Table S**4. Correlation Between CD133 and GPR87 Expression Levels in HCC Patients without Intrahepatic Metastasis and Their Clinicopathologic Characteristics.

| Clinical Pathology | | CD133 | | | GPR87 | | |
| --- | --- | --- | --- | --- | --- | --- | --- |
|  |  | Negative  (%) | Positive  (%) | *P* Value | Negative  (%) | Positive  (%) | *P* Value |
| Gender | Male | 26(19.5) | 107(80.5) | 0.062 | 75(56.4) | 58(43.6) | 0.263 |
|  | Female | 10(35.7) | 18(64.3) |  | 19(67.9) | 9(32.1) |  |
| Age | ≤50 | 30(28.6) | 75(71.4) | 0.011^*^ | 60(57.1) | 45(42.9) | 0.568 |
|  | >50 | 6(10.9) | 49(89.1) |  | 34(61.8) | 21(38.2) |  |
| AFP (ng/mL) | ≤20 | 14(24.1) | 44(75.9) | 0.521 | 32(55.2) | 26(44.8) | 0.603 |
|  | >20 | 20(19.8) | 81(80.2) |  | 60(59.4) | 41(40.6) |  |
| HBsAg | Absent | 8(26.7) | 22(73.3) | 0.522 | 17(56.7) | 13(43.3) | 0.873 |
|  | Present | 27(21.3) | 100(78.7) |  | 74(58.3) | 53(41.7) |  |
| HBeAg | Absent | 28(22.6) | 96(77.4) | 0.709 | 67(54.0) | 57(46.0) | 0.120 |
|  | Present | 7(25.9) | 20(74.1) |  | 19(70.4) | 8(29.6) |  |
| antiHBe | Absent | 20(24.4) | 62(75.6) | 0.737 | 57(69.5) | 25(30.5) | 0.001^*^ |
|  | Present | 15(22.1) | 53(77.9) |  | 29(42.6) | 39(57.4) |  |
| antiHBc | Absent | 6(17.6) | 28(82.4) | 0.373 | 23(67.6) | 11(32.4) | 0.167 |
|  | Present | 29(25.0) | 87(75.0) |  | 63(54.3) | 53(45.7) |  |
| antiHCV | Absent | 21(28.4) | 53(73.0) | 0.164 | 37(50.0) | 37(50.0) | 0.655 |
|  | Present | 0(0.0) | 5(100.0) |  | 3(60.0) | 2(40.0) |  |
| Histological grade | Ⅰ-Ⅱ | 23(22.1) | 81(77.9) | 0.920 | 62(59.6) | 42(40.4) | 0.669 |
|  | Ⅲ-Ⅳ | 13(22.8) | 44((77.2) |  | 32(56.1) | 25(43.9) |  |
| Tumor size (cm) | ≤5 | 16(21.3) | 59((78.7) | 0.988 | 46(61.3) | 29(38.7) | 0.592 |
|  | >5 | 18(21.4) | 66(78.6) |  | 48(57.1) | 36(42.9) |  |
| Cirrhosis | Absent | 8(21.6) | 29(78.4) | 0.902 | 20(54.1) | 17(45.9) | 0.543 |
|  | Present | 28(22.6) | 96(79.8) |  | 74(59.7) | 50(40.3) |  |

*P* value represents the probability from a chi-square test for CD133 and GPR87 expression levels between variable subgroups.

**P* < 0.05.

**Abbreviations:** GPR87, G protein-coupled receptor 87; HCC, hepatocellular carcinoma;

AFP, alpha-fetoprotein; HBsAg, hepatitis B surface antigen; antiHBs, anti-hepatitis B surface antibody; HBeAg, hepatitis B e antigen; antiHBe, anti-hepatitis B e antibody; antiHBc, anti-hepatitis B core antibody; antiHCV, anti-hepatitis C virus antibody.
