# Supplementary material for: G Protein-Coupled Receptor 87 (GPR87) Promotes the Growth and Metastasis of CD133+ Cancer Stem-Like Cells in Hepatocellular Carcinoma
Source: PLoS One. 2013 Apr 10;8(4):e61056. doi: 10.1371/journal.pone.0061056 (PMC3622685; doi:10.1371/journal.pone.0061056)
Supplement: Table S5 — Correlation Between CD133 and GPR87 Expression in HCC Tissues without Intrahepatic Metastasis. (DOC) [file pone.0061056.s011.doc]

**Table S5. Correlation Between CD133 and GPR87 Expression in HCC Tissues without Intrahepatic Metastasis.**

|  |  | GPR87 Scores | |
| --- | --- | --- | --- |
|  |  | 0(%) | 1(%) |
| CD133 Scores | 0 | 25(26.6) | 69(73.4) |
|  | 1 | 11(16.4) | 56(83.6) |

R=0.120, *P* = 0.128.
